# In Situ SR-XPS Observation of Ni-Assisted Low-Temperature Formation of Epitaxial Graphene on 3C-SiC/Si

**DOI:** 10.1186/s11671-015-1131-9

**Published:** 2015-10-26

**Authors:** Mika Hasegawa, Kenta Sugawara, Ryota Suto, Shota Sambonsuge, Yuden Teraoka, Akitaka Yoshigoe, Sergey Filimonov, Hirokazu Fukidome, Maki Suemitsu

**Affiliations:** Research Institute of Electrical Communications, Tohoku University, Sendai, 980-8577 Japan; JAEA, Kouto, Sayo-cho, Sayo-gun, Hyogo 679-5148 Japan; Department of Physics, Tomsk State University, Tomsk, Russia; JST-CREST, Tokyo, Chiyoda, Tokyo 107-0075 Japan

**Keywords:** Graphene, AR-XPS, 3C-SiC, Ni silicide, Ni carbide

## Abstract

Low-temperature (~1073 K) formation of graphene was performed on Si substrates by using an ultrathin (2 nm) Ni layer deposited on a 3C-SiC thin film heteroepitaxially grown on a Si substrate. Angle-resolved, synchrotron-radiation X-ray photoemission spectroscopy (SR-XPS) results show that the stacking order is, from the surface to the bulk, Ni carbides(Ni_3_C/NiC_x_)/graphene/Ni/Ni silicides (Ni_2_Si/NiSi)/3C-SiC/Si. In situ SR-XPS during the graphitization annealing clarified that graphene is formed during the cooling stage. We conclude that Ni silicide and Ni carbide formation play an essential role in the formation of graphene.

## Background

Graphene, a single layer of sp^2^-bonded carbon atoms arranged in a honeycomb lattice, has attracted much attention as a promising material in electronics and photonics. This is mainly due to its giant carrier mobility (200,000 cm^2^/Vs) [1], high thermal conductivity, and high mechanical strength [2–4], as well as to its adaptability in integrated planar technology. Various methods of the graphene formation have been reported, including mechanical exfoliation of graphite [1], CVD on catalytic metals [5–8], and epitaxial graphene formation (EG) on hexagonal (4H- and 6H-) SiC bulk wafers [9–11]. Among these proposed methods, the EG method is considered preferable in that it not only allows growth of large-area graphene but also yields a well-defined graphene/substrate interface. This is because the method consists entirely of conventional thermal processes and is thus free from transfer of graphene onto substrates, unlike mechanical exfoliation and CVD methods, which sometimes causes the onset of wrinkles in graphene and residual impurities at the interface. The challenge of the EG method, however, is the cost of high-quality SiC bulk wafers and the use of the SiC wafer itself, which forms a barrier for graphene to be adapted into Si technology.

In this context, Suemitsu et al. developed a so-called graphene-on-silicon (GOS) technology, in which EG is formed on a 3C-SiC thin film heteroepitaxially grown on a Si wafer [12–15]. The merit of the GOS method lies, first of all, in the use of large-area, cost-effective Si wafers, which makes this technology even more practical in Si technology. Moreover, GOS provides a good control over the structure of the graphene/SiC interface by simply changing the crystallographic orientation of the Si surface [16–18]. This allows to deliberately include or exclude the buffer layer at the graphene/SiC interface and thereby to control the electronic and optical properties of graphene as well. Several reports on the device fabrication based on GOS already exist [19–23].

A graphitization temperature of ~1473 K or higher, however, is still too high to be fully compatible with Si technology. Several attempts have been made to reduce the graphitization temperature of GOS, including the addition of a small amount of oxygen [24] or monosilane [25] into the vacuum used for the graphitization annealing. Although these methods succeeded in forming epitaxial graphene at ~1273 K, the graphene quality was much lower than that of the conventional GOS and EG methods. Recently, low-temperature (~973 K) formation of EG on SiC bulk crystals has been reported by Escobedo-Cousin et al. [26], who utilized a solid-phase chemical reaction between SiC and Ni. In fact, the formation of a “thin graphite layer” at low temperatures had been reported for ohmic-contact fabrication on SiC using Ni, well before [27] or at around the same time [28] of the rise of the graphene fever in the mid-2000s. Although these earlier studies suggested nickel silicide formation at the Ni/SiC interface and subsequent emission of excessive C atoms as a possible mechanism of the graphite formation, little is known about the details of the process. It has also been unclear whether this method is applicable to the GOS process, i.e., on 3C-SiC films. Here, we report the first application of this Ni-assisted graphene formation to GOS. Moreover, solid-phase reactions during heating/annealing/cooling procedures have been investigated in detail by using in situ synchrotron-radiation X-ray photoelectron spectroscopy (SR-XPS). Angle-resolved (AR) analysis was also utilized to clarify the stacking order of the layers. As a result, we demonstrate that this Ni-assisted method is equally applicable to 3C-SiC thin films (GOS), where graphene forms at temperatures less than 1273 K. We also clarify the role of Ni/SiC reactions, in which not only the formation of Ni silicide but also Ni carbonization is suggested as a key process in the formation of graphene.

## Methods

An n-type Si(111) wafer was cut into pieces to form specimens with a size of 7 × 7 mm^2^. The sample fabrication consists of four stages: (a) growth of 3C-SiC thin (~100 nm) films using gas-source molecular-beam epitaxy (GSMBE), (b) ultrathin (~2-3 nm) Ni film deposition using electron beam, (c) annealing at temperatures 973–1173 K for 30 min, and (d) cooling down to 373 K at a rate of 12.5 K/min. In GSMBE, monomethylsilane (MMS: CH_3_SiH_3_) with a pressure of 10^−2^ Pa was used as the single source of silicon and carbon with a typical substrate temperature of 1323 K [29]. Graphitization annealing was conducted in ultrahigh vacuum (UHV) by heating the samples radiatively from the back with a graphite heater. Graphene formation was confirmed by Raman scattering spectroscopy. As for the analysis on reactions at the Ni/3C-SiC interface during heating/annealing/cooling of the samples, in situ SR-XPS observations were conducted at the BL23SU surface chemistry end-station [30] in the SPring-8 facilities using 650 eV photons. Angle-resolved (AR) observations were also conducted to obtain information on the order of the stacking layers. Since the photoelectrons that escape the surface at angles in the range of ±8° around the line-of-sight direction from the surface to the analyzer were integrally collected in the present AR-XPS, only relative intensities between components were utilized in the analysis to avoid complexity.

## Results and Discussion

### Confirmation of Graphene Formation

**Fig. 1:**
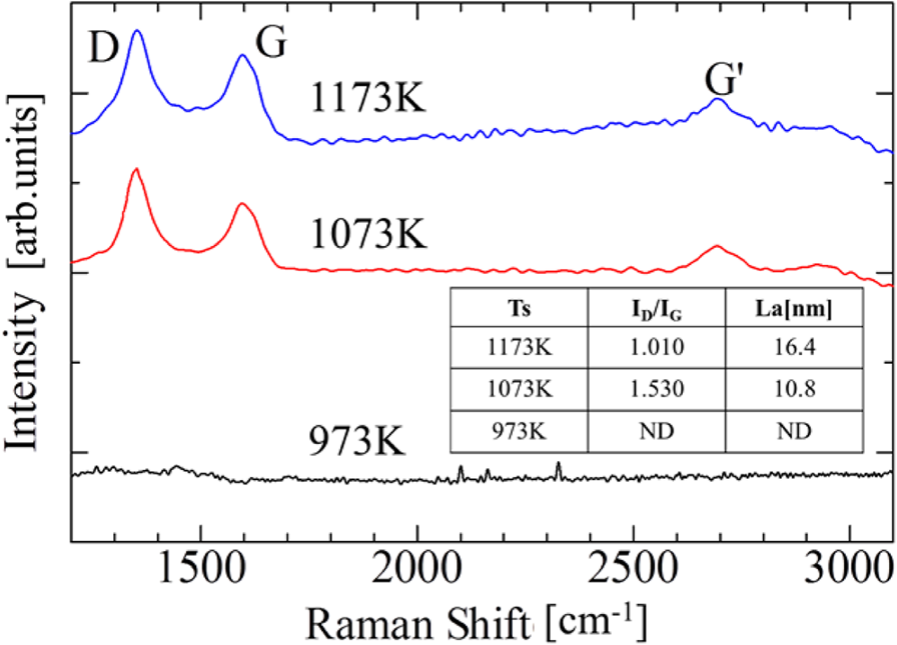
Raman spectra of the graphene. The grain size of the graphene, estimated from the intensity ratio *I*_D_/*I*_G_, is shown in the table

Figure 1 shows the Raman spectra of the samples after annealing at three different temperatures. At temperatures 1073 and 1173 K, the three major fundamental vibration modes of graphene, i.e., G(~1600 cm^−1^), G’(~2700 cm^−1^), and D(~1360 cm^−1^) appear, which proves the graphene formation at these low temperatures. The grain size of graphene (*L*_*a*_) is obtained from the intensity ratio between the D and G bands as

$${L}_a\left(\mathrm{nm}\right)=\frac{560}{E_{\mathrm{laser}}^4}{\left(\frac{I_{\mathrm{D}}}{I_{\mathrm{G}}}\right)}^{-1},$$

where *E*_laser_ is the energy of the excitation laser in electron volt, and *I*_D_ and *I*_G_ are the intensities of the D and G bands, respectively [31]. At 1173 K, the estimated grain size is larger than that at 1073 K, as shown in the inset of Fig. 1.

### The Mechanism of Ni-Assisted Graphene Formation

#### Determination of the Stacking Order of the Layers

**Fig. 2:**
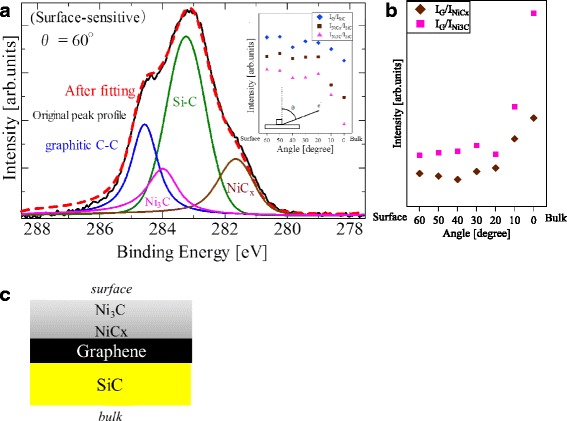
C 1s core-level SR-XPS spectrum. **a** Surface-sensitive SR-XPS spectrum obtained at the detection angle of 60° with respect to the surface normal. *Inset* is the relative peak intensity of graphene and Ni carbides (Ni_3_C and NiC_x_) normalized by that of SiC. **b** SR-XPS peak intensity ratio of graphene normalized by the Ni carbides (Ni_3_C and NiC_x_). **c** A model for the order of the stacking layers

Where is the graphene in the multi-layered Ni/SiC/Si structure? To answer this question, we conducted an AR-XPS analysis. Figure 2a shows the C 1s core-level spectrum obtained in a surface-sensitive mode with the detection angle of 60° from the surface normal. The Ni carbide components such as Ni_3_C (~284 eV) and NiC_*x*_ (~282 eV, *x* > 0.33) [32] appear in the spectrum together with the ones from 3C-SiC (~283.5 eV) and graphene (~284.5 eV) [33]. The escape depths *λ* of the photoelectrons from Ni-C, Ni, and Ni-Si layers are in the order of 1–1.7 nm, which decreases down to 0.5–0.8 nm in the surface-sensitive measurement at *θ* = 60°. Although this value is less than the thickness of the Ni layer (~2 nm), we here observe a strong SiC component in the C 1s spectrum. This result can be related to the fact (a) that XPS measurement substantially detects photoelectrons from the surface up to 3λ in depth and (b) that our XPS setup integrally collects the photoelectrons that leave the surface at angles in the range of ±8° around the line-of-sight direction from the surface to the analyzer. The inset shows the detection-angle dependence of the peak intensities normalized by that of the SiC peak. The graphene and the Ni-carbide peaks show higher intensities in the surface-sensitive observations, indicating the formation of these layers on top of the SiC film. Figure 2b shows the relative peak intensity of graphene against Ni_3_C and NiC_x_. They are higher in the bulk-sensitive observations, and this tendency is pronounced in the I_G_/I_Ni3C_ ratio. It is therefore concluded that the graphene layer is located beneath the Ni carbide layers, and the Ni_3_C layer is located above the C-rich NiC_x_ (*x* > 0.33) layer (Fig. 2c).

**Fig. 3:**
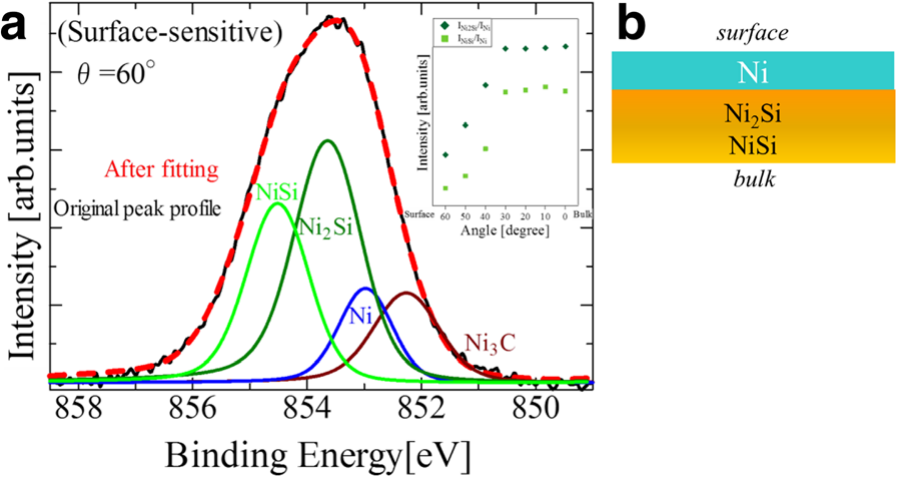
Ni 2p_3/2_ core-level spectrum of SR-XPS. **a** Surface-sensitive SR-XPS spectrum obtained at the detection angle of 60° with respect to the surface normal. *Inset* is the relative peak intensity of Ni silicides (Ni_2_Si and NiSi) normalized by that of Ni. **b** A model for the stacking order between Ni and Ni silicide layers

Figure 3a shows the Ni 2p_3/2_ core-level spectrum obtained in a surface-sensitive mode with the detection angle of 60° from the surface normal. The Ni silicide components such as Ni_2_Si (~854 eV) and NiSi (~854.9 eV) and the Ni carbide component Ni_3_C (852.4 eV) [34] appear in the spectrum as well as the one from Ni (~853 eV). The inset shows the detection-angle dependence of the Ni silicide peak intensities normalized to that of the Ni peak. They show higher intensities in the bulk-sensitive observations, indicating that the Ni silicides are located beneath the Ni layer. It is also noted that the relative intensity of Ni_2_Si to NiSi increases in the surface-sensitive observations, indicating that the Ni-rich Ni_2_Si silicide is on top of the Si-rich NiSi silicide. We therefore conclude that the Ni and Ni-silicide layers are stacked, from the surface to the bulk, in the order of Ni/Ni_2_Si/NiSi (Fig. 3b).

**Fig. 4:**
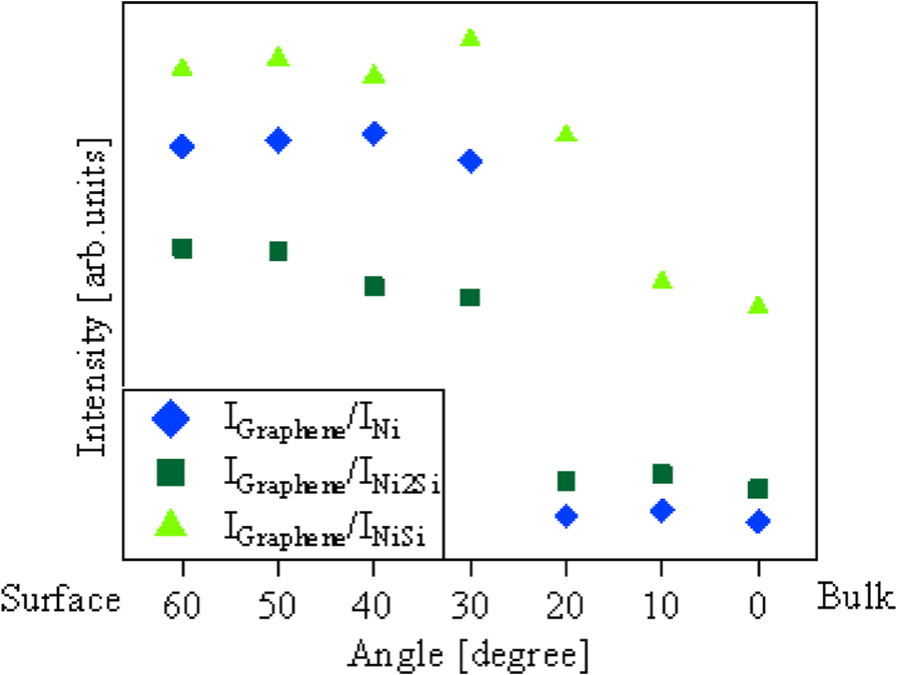
Graphene peak intensity of C 1s core-level SR-XPS spectra normalized by Ni and Ni silicide peak intensity of Ni 2p core-level SR-XPS spectra

**Fig. 5:**
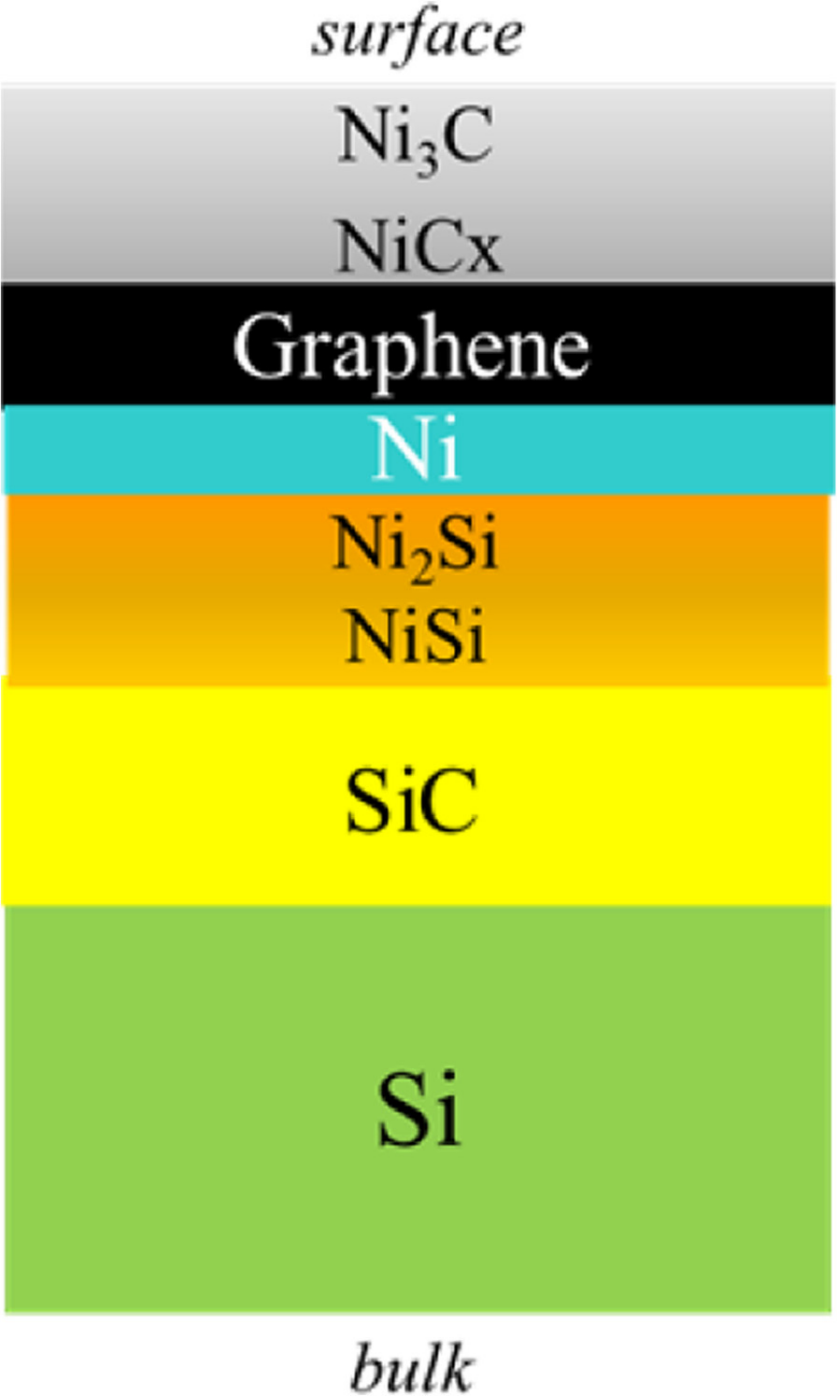
The estimated order of the stacking layers from C 1s and Ni 2p core-level angle-resolved SR-XPS

Figure 4 shows the detection-angle dependence of the relative intensity of the graphene peak normalized by that of Ni and Ni silicides. It shows higher intensities in the surface-sensitive observations. It is thus concluded that graphene is formed on top of the Ni and Ni silicide films. The inflection points seen at 20-30° are currently related to a possible abruptness at interfaces, but its detailed understandings are open to future studies. By summing up all the AR-XPS results, we conclude that the order of the stacking is as shown in Fig. 5.

#### In Situ SR-XPS Observation of the Graphene Formation

**Fig. 6:**
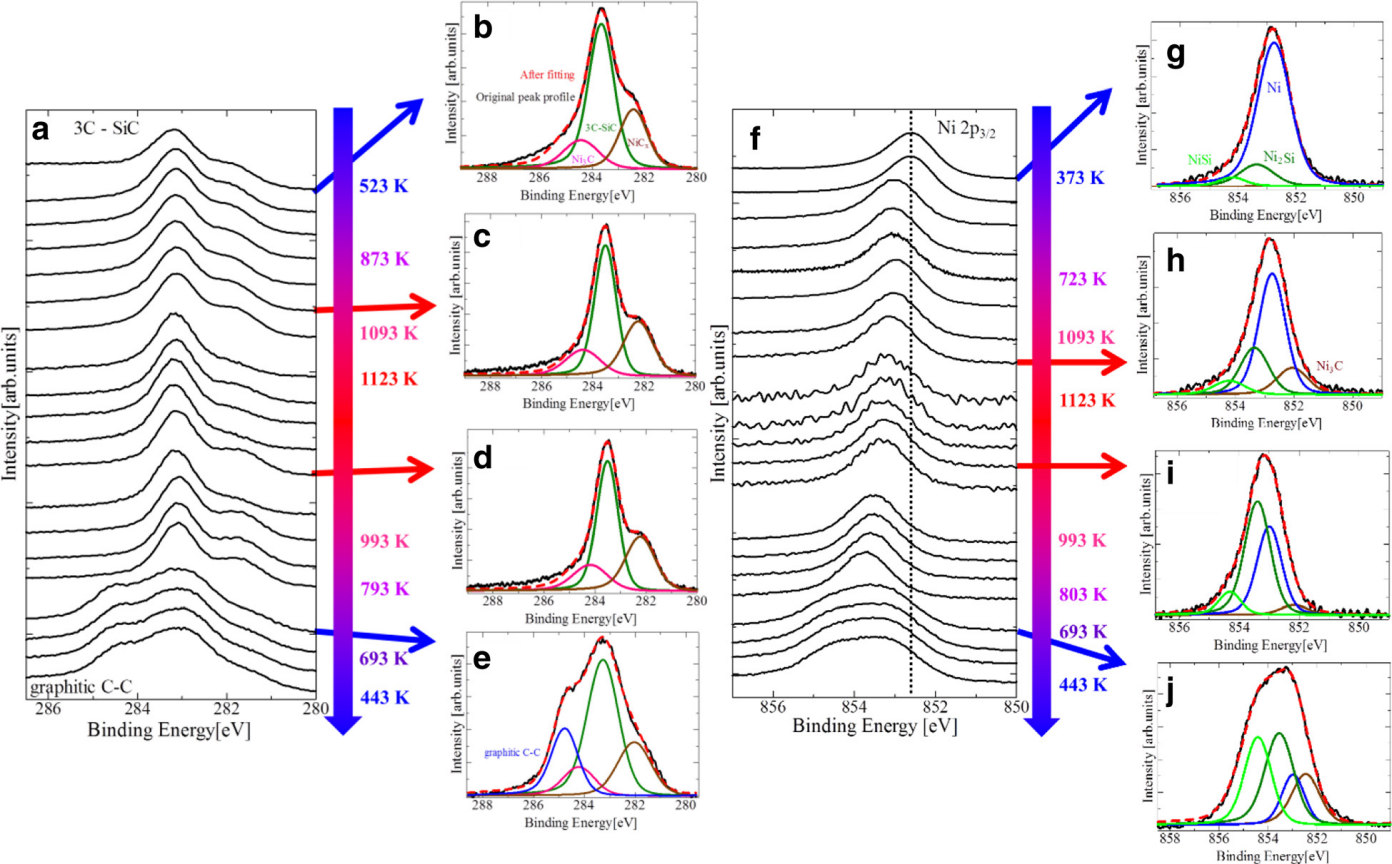
In situ SR-XPS spectra in the reaction process. **a**–**e** C 1s core-level SR-XPS spectra. **f**-**j** Ni 2p_3/2_ core-level SR-XPS spectra

In order to clarify the role of reactions between Ni and SiC on the formation of graphene, in situ SR-XPS was conducted during heating, annealing (1123 K for 30 min), and cooling of the sample (Fig. 6a). The Ni carbide components, Ni_3_C and NiC_x_ as well as that of 3C-SiC, appear in the spectra in the early stage of the heating. Graphene component, on the other hand, appears only in the cooling stage (Fig. 6e). In the Ni 2p_3/2_ core-level SR-XPS spectra (Fig. 6f), the peak shifts to higher binding energies during the heating/annealing procedures. This blue shift could mean either formation of Ni silicides [34] or Ni carbides [35], or both, and is shown to be accelerated during the cooling stage. Specifically, the Ni-rich silicide Ni_2_Si first forms during heating and NiSi and Ni_3_C form during annealing and cooling.

From these time evolutions of both C 1s and Ni 2p core-level peaks, we conclude that part of the 3C-SiC film is decomposed due to reactions with Ni to form Ni silicides and Ni carbides, and the excess C atoms created by this decomposition are used to form graphene. Of the Ni silicide and carbide formations, the former should be by far the major reaction path because the formation enthalpy for Ni silicide is negative (−80 kJ/mol) while that for Ni carbide is positive (30 kJ/mol) [35–37]. Kurimoto et al. [27] also suggested Si diffusion into the Ni layer to form Ni_2_Si as a major reaction path for the graphite layer formation. As for graphene, Cao et al. [28] have reported the formation of an amorphous C layer followed by its constituents diffusing toward the surface as a possible mechanism. A new finding in this study, however, is that part of the excess C atoms is used for the formation of Ni carbides as well, as shown in Figs. 2 and 6. Since the formation of Ni carbide is prohibited at normal pressures for our annealing temperatures [36, 38], the present observation of Ni carbide should be related to the fact that our annealing was conducted under a non-equilibrium UHV condition, unlike the ohmic-contact fabrication process conducted at normal pressures. In any case, the rest of the C atoms are dissolved in Ni as a result of the high solubility (~2.7 %) in Ni at high temperatures (>1000 K). Upon cooling, these dissolved C atoms are prone to segregate to the surface to decrease the surface energy [39, 40]. In the present case, however, the surface of Ni is already covered with Ni carbide layers, whose solubility for C atoms is too low (~0.01 %) [41] either to accommodate them therein or to let them diffuse through the layer to the surface. This is understood to be the reason why excess C atoms take the form of graphene at the interface between Ni and Ni carbides.

## Conclusions

In order to verify the Ni-assisted graphene formation on the SiC thin films on Si substrates and to clarify its growth mechanism, we conducted Raman and in situ SR-XPS analysis. We have successfully demonstrated that graphene actually forms on a 3C-SiC thin film at a temperature as low as 1073 K with the aid of the Ni layer. Angle-resolved SR-XPS proved that the stacking order, from the top to the bottom, is Ni carbides (Ni_3_C/NiC_x_)/graphene/Ni/Ni silicides (Ni_2_Si/NiSi)/SiC/Si. In situ SR-XPS during heating/annealing/cooling procedures has proved that Ni silicides and Ni carbides form during heating/annealing periods while graphene forms only in the cooling period. It is suggested that excess C atoms are liberated during heating/annealing along with the formation of Ni silicides, and part of these excess C atoms form into graphene. Graphene is formed during cooling because the C atom solubility in Ni decreases with decreasing temperature. The Ni carbide layers are suggested to act as a good capping layer for graphene formation as well as a C source through its decomposition during cooling, which is favored because of its exothermic nature of the reaction.

The obtained graphene quality is still mediocre (D/G Raman ratio ~1.0), and there is a lot of room for improvement toward low-temperature formation of graphene. Moreover, graphene produced by this method grows on a non-crystalline Ni layer, not directly on the SiC crystal, which cannot be called “epitaxial graphene” in its true meaning of the term. Despite these limitations, the present findings, especially that on the role of Ni carbide formation, will greatly contribute to the improvement of Ni-assisted graphene formation on Ni/SiC as well as ohmic-contact formation for both graphene and SiC devices.
